# Completely detached mechanical aortic valve prosthesis stuck to the aortic arch in a patient with Behçet’s disease

**DOI:** 10.1186/s40792-022-01506-6

**Published:** 2022-07-29

**Authors:** Haruhiro Nagase, Takaya Hoashi, Ayumu Masuoka, Kentaro Hotoda, Koichi Toda, Akihiro Yoshitake, Takaaki Suzuki

**Affiliations:** 1 Pediatric Cardiac Surgery, Saitama Medical University International Medical Center, 1397-1 Yamane, Hidaka, Saitama 350-1298 Japan; 2 Pediatric Cardiology, Saitama Medical University International Medical Center, 1397-1 Yamane, Hidaka, Saitama 350-1298 Japan; 3 Cardiovascular Surgery, Saitama Medical University International Medical Center, 1397-1 Yamane, Hidaka, Saitama 350-1298 Japan

**Keywords:** Behcet's disease, Aortic valve replacement, Valve detachment

## Abstract

**Background:**

Although detachment of the implanted valve prosthesis was a well-known complication in patients with Behçet’s disease, complete detachment of an aortic bileaflet valve prosthesis has never been reported.

**Case presentation:**

An 18-year-old boy with Behçet’s disease (HLA-A26 positive) who had previously undergone aortic valve replacement with an 18-mm ATS-Advanced Performance (ATS-AP) valve (ATS Medical, Inc., Minneapolis, MN) at the age of 12 years, presented sudden-onset general fatigue and was emergently transferred to the regional hospital. Chest X-ray showed displacement of the implanted mechanical valve. An echocardiogram revealed mobile valve prosthesis and severe aortic regurgitation. Just before leaving for our hospital for surgical treatment, a completely detached valve prosthesis was floating in the ascending aorta. On arrival, the valve prosthesis was stuck to the transverse arch. Emergent removal of the previous mechanical valve from the aortic arch and redo aortic valve replacement with a 24-mm ATS-AP valve were performed under total circulatory arrest. Infectious endocarditis was denied by histopathological examination. The patient was back to the intensive care unit with extracorporeal membrane oxygenation support, which was successfully decannulated 5 days later.

**Conclusions:**

This was the first report of a patient with Behçet’s disease who encountered a complete detachment of implanted aortic valve prosthesis. The patient could be rescued by emergent surgery.

## Introduction

Although detachment of the implanted valve prosthesis was a well-known complication in patients with Behçet’s disease, complete detachment of an aortic bileaflet valve prosthesis has never been seen. Herein, we reported a young gentleman with Behçet’s disease who encountered a complete detachment of implanted aortic valve prosthesis 6 years later.

## Case presentation

An 18-year-old boy with Behçet’s disease (HLA-A26 positive) who had previously undergone aortic valve replacement with an 18-mm ATS Open Pivot AP360 mechanical aortic valve prosthesis (ATS Medical, Inc, Minneapolis, MN) at the age of 12 years, presented sudden-onset general fatigue and was emergently transferred to the regional hospital. Since initial aortic valve replacement, 10 mg a day of prednisolone sodium succinate, 12 mg once weekly of methotrexate, 750 mg every 4–6 weeks of infliximab, and 35 mg every 2 weeks of alendronate sodium hydrate had been administered.

**Fig. 1:**
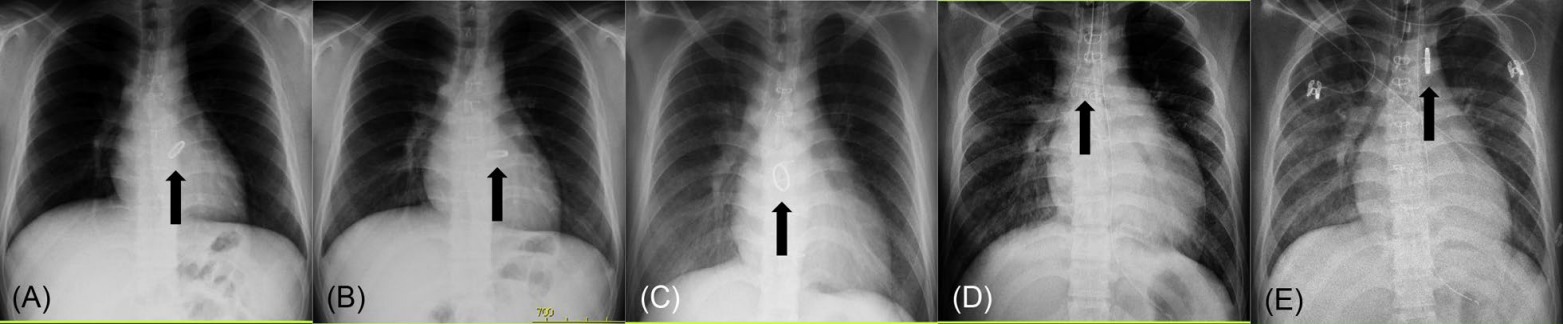
Chest X-ray findings 3 months ago (**A**), 1 week ago (**B**), at emergent admission to the regional hospital (**C**), at leaving for our hospital (**D**), and at arriving at our hospital (**E**). Black arrows indicating mechanical valve

Chest X-ray showed displacement of the implanted mechanical valve (Fig. 1A, C). From a retrospective point of view, slight displacement of the valve prosthesis had been already confirmed 1 week ago at the scheduled follow-up (Fig. 1B). An echocardiogram revealed mobile valve prosthesis and severe aortic regurgitation. Just before leaving for our hospital for surgical treatment, a completely detached valve prosthesis was floating in the ascending aorta (Fig. 1C).

**Fig. 2:**
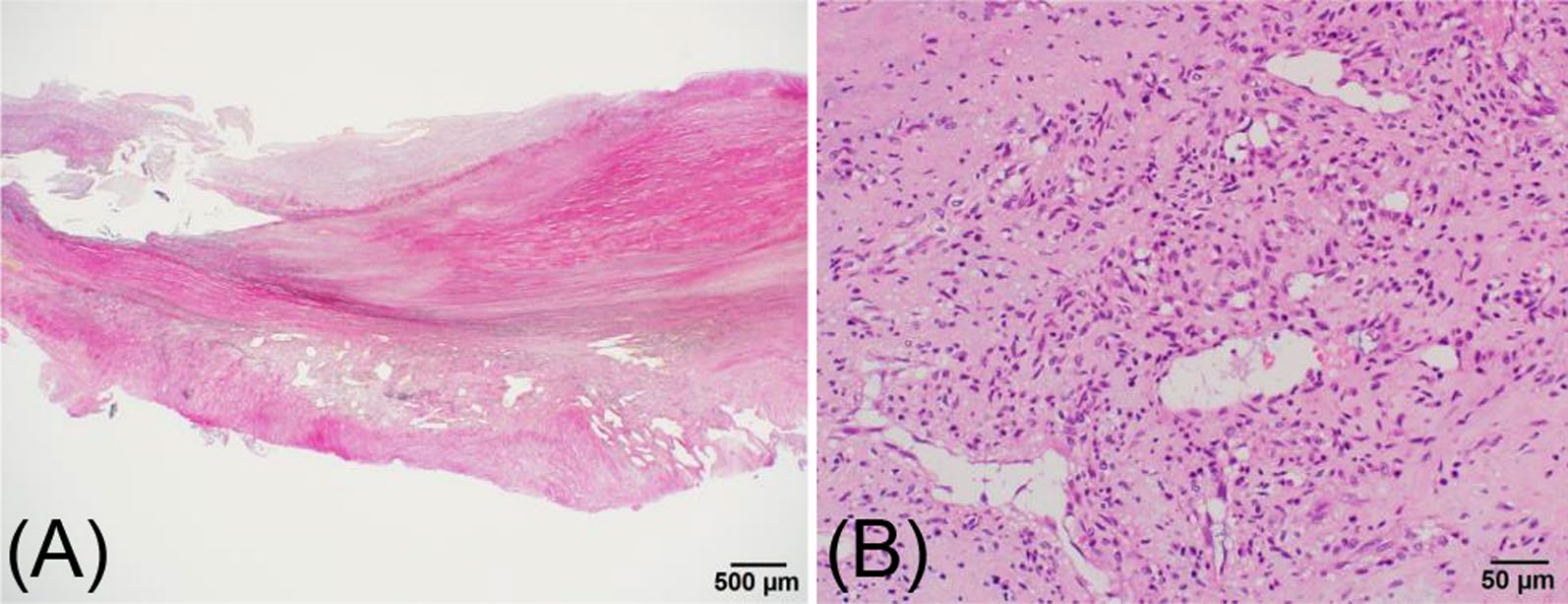
**A** Photomicrograph (×2, scale bar = 500 μm) by elastica van Gieson staining showed fibrous tissue with granulation and degeneration. **B** Photomicrograph (×200, scale bar = 50 μm) by hematoxylin and eosin staining showed granulation tissue and degenerated collagen fibers, with microvasculature, fibroblast, and inflammatory cells

On arrival, the valve prosthesis was stuck to the transverse arch (Fig. 1D). Emergent removal of the previous mechanical valve from the aortic arch and redo aortic valve replacement were performed under total circulatory arrest. After a removal of fibrous tissues around annulus on where previous valve prosthesis was placed, 14 pairs of pledgeted 2–0 polyester terephthalate braided, non-everting mattress suture from deep subannular left ventricular endocardium to the aortic wall just above the true annulus were placed to prevent recurrent detachment of implanted valve prosthesis. After that, a newly fashioned 24-mm ATS Open Pivot AP360 valve could be implanted in the supra-annular position without any annular enlargement procedures. The patient was back to the intensive care unit with extracorporeal membrane oxygenation support. Histopathology of the surrounding tissue around a previous valve prosthesis showed no bacterial infection sign, then only inflammatory change was noted (Fig. 2A, B).

Extracorporeal membrane oxygenation was successfully decannulated 5 days later. Intra-arterial balloon pump and hemodialysis could be weaned off on post-operative day 8. Intra-cranial computed tomography was performed on post-operative day 29, which revealed the hypoxic ischemic change in bilateral basal ganglia and multiple old microinfarctions. Left ventricular dysfunction has remained since then, but catecholamine support could be discontinued 2 months later. Now 3 months have been passed since the emergent surgery. He was still supported by phosphodiesterase III inhibitor, but no longer supported by mechanical ventilator. Left ventricular end-diastolic diameter and ejection fraction were 43 mm and 25%, respectively.

Sixty mg a day of soluble predonine had been initiated since post-operative day 13, which then was gradually tapered to 30 mg a day for 3 months.

## Discussion

Detachment of implanted aortic valve prostheses is a well-known complication after aortic valve replacement in patients with Behçet’s disease [1, 2]. As well as an aggressive immunosuppression therapy with oral steroid administration, several technical modifications at initial valve implantation have been recently reported to prevent later valve prosthesis dehiscence [3–6].

Although complete detachment of valve prosthesis was reported in an autopsy case that was complicated by infective endocarditis [7], complete detachment of bileaflet mechanical aortic valve prosthesis in patients with Behçet’s disease has never been reported. Behçet’s disease is known to be typically associated with HLA-B51, however, a previous study showed that HLA-B51 positive did not result in frequent manifestations or severe disease course [8]. Rather, the association of atypical class I HLA antigen, such as HLA-A26 (which is positive in this patient), might be affected. Or, annular dilatation may progress during interstage period from initial aortic valve replacement to this sudden complete detachment, because mechanical valve size up from 18 to 24 mm was possible at this emergent surgery without any annular enlargement procedures.

Emergent surgery was performed just after arriving at our center. Fortunately, previously detached implanted valve prosthesis could be easily retrieved via distal ascending aortotomy under total circulatory arrest without injuring the surrounding structures. A new mechanical valve was implanted at the supra-annular position with non-everting mattress suture from deep left ventricular side with pledgets, according to a concept of previous report but subannular ring was not used [3]. Left ventricular dysfunction remained, but catecholamine support could be discontinued 2 months later. From a retrospective point of view, slight displacement of the valve prosthesis had been already confirmed 1 week before emergent admission, at scheduled follow-up (Fig. 1B). Although valve implantation technique was modified and stronger immunosuppression therapy has been initiated, care should be taken not to overlook a repeat detachment of implanted valve prosthesis.

## Conclusions

This was the first report of a patient with Behçet’s disease who encountered a complete detachment of implanted aortic valve prosthesis. The patient could be rescued by emergent surgery.

## Data Availability

No additional data.
